# {μ-1,5-Bis[(*E*)-1-(2-pyrid­yl)ethyl­idene]carbonohydrazidato(1−)}bis­[chlorido­methano­lcopper(II)] perchlorate

**DOI:** 10.1107/S1600536809040471

**Published:** 2009-10-10

**Authors:** Wei Huang

**Affiliations:** a Anhui Key Laboratory of Functional Coordination Compounds, School of Chemistry and Chemical Engineering, Anqing Teachers College, Anqing, 246011 Anhui, People’s Republic of China

## Abstract

The title dinuclear copper complex, [Cu_2_(C_15_H_15_N_6_O)Cl_2_(CH_3_OH)_2_]ClO_4_, was prepared by the reaction of copper(II) chloride with bis­[1-(2-pyrid­yl)ethyl­idene]carbonohydrazide in the presence of sodium perchlorate in a methanol solution. It features a mono-deprotonated bis-tridentate ligand, which coordinates to two independent Cu^II^ ions, one of which is coordinated by pyridyl N, hydrazyl N and carbonyl O atoms. The second Cu^II^ ion is coordinated by the pyridyl N and two hydrazyl N atoms from different hydrazyl groups. The coordination environments of both Cu^II^ ions are completed by a chloride ion and a methanol mol­ecule. The dihedral angle between the pyridyl groups is 27.46 (10)°. The crystal packing is stabilized by O—H⋯O(perchlorate), O—H⋯Cl and N—H⋯Cl hydrogen bonding.

## Related literature

For the definition of the distortion parameter, see: Addison *et al.* (1984 ▶).
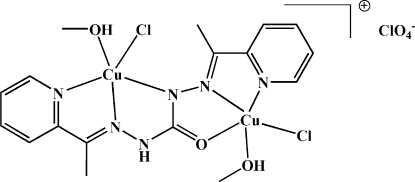


## Experimental

### 

#### Crystal data

[Cu_2_(C_15_H_15_N_6_O)Cl_2_(CH_4_O)_2_]ClO_4_
*M* *_r_* = 656.84
Orthorhombic, 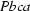

*a* = 8.0319 (3) Å
*b* = 16.6784 (6) Å
*c* = 37.3492 (13) Å
*V* = 5003.3 (3) Å^3^
*Z* = 8
Mo *K*α radiation
μ = 2.07 mm^−1^
*T* = 298 K
0.18 × 0.16 × 0.12 mm

#### Data collection

Bruker SMART APEXII CCD area-detector diffractometer
Absorption correction: multi-scan (*SHELXTL*; Sheldrick, 2008 ▶) *T*_min_ = 0.675, *T*_max_ = 0.783
30236 measured reflections
5692 independent reflections
4016 reflections with *I* > 2σ(*I*)
*R*_int_ = 0.064

#### Refinement

*R*[*F* ^2^ > 2σ(*F* ^2^)] = 0.048
*wR*(*F* ^2^) = 0.142
*S* = 1.04
5692 reflections
318 parameters
H-atom parameters constrained
Δρ_max_ = 0.90 e Å^−3^
Δρ_min_ = −0.67 e Å^−3^

### 

Data collection: *SMART* (Bruker, 1997 ▶); cell refinement: *SAINT* (Bruker, 1997 ▶); data reduction: *SAINT*; program(s) used to refine structure: *SHELXTL* (Sheldrick, 2008 ▶); molecular graphics: *SHELXTL*; software used to prepare material for publication: *SHELXTL*.

## Supplementary Material

Crystal structure: contains datablocks I, global. DOI: 10.1107/S1600536809040471/fi2081sup1.cif

Structure factors: contains datablocks I. DOI: 10.1107/S1600536809040471/fi2081Isup2.hkl

Additional supplementary materials:  crystallographic information; 3D view; checkCIF report

## Figures and Tables

**Table 1:** Hydrogen-bond geometry (Å, °)

*D*—H⋯*A*	*D*—H	H⋯*A*	*D*⋯*A*	*D*—H⋯*A*
N3—H3*B*⋯Cl2^i^	0.86	2.91	3.766 (4)	176
O1*W*—H1*W*⋯Cl1^ii^	1.01	2.31	3.205 (4)	147
O2*W*—H2*W*⋯O7^iii^	1.00	1.90	2.855 (6)	159

Symmetry codes: (i) 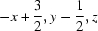; (ii) 
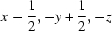
; (iii) 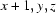.

**Fig. 1.**
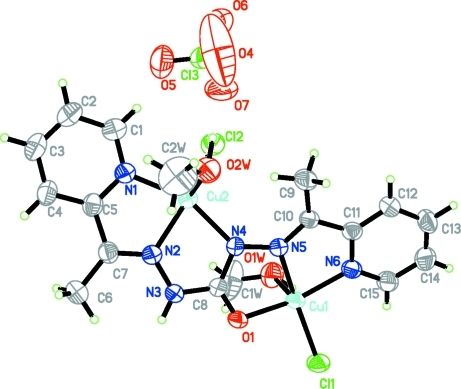
The dinuclear cation and the perchlorate anion in the structure of the title compound. Thermal ellipsoids are drawn at the 50% level.

**Fig. 2.**
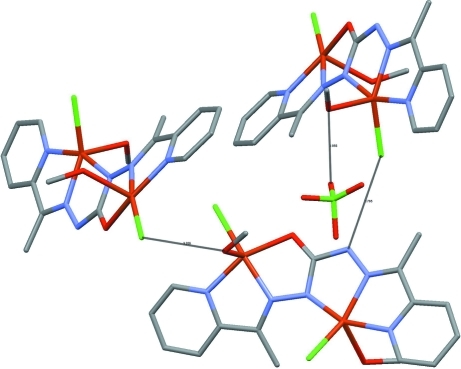
The hydrogen bonding pattern in the title compound.

## supplementary crystallographic information

### Comment

Recent research has witnessed considerable interest in the development of new multidentate ligands. Polyamine tripod ligands containing appropriate binding sites and shape may be designed to form special topological structures. The molecular topology of the host molecule can be synthetically modulated to bind many different chemical species from transition metals to lanthanide ions. To this purpose, the title compound was synthesized and structurally characterized. It features a dinuclear copper(II) complex assembled from one mono-deprotonated, bis-tridentate ligand and two distinct copper(II) ions. The coordination of Cu1 is achieved by pyridyl-N, hydrazine-N (deprotonated hydrazine group) and carbonyl-O, Cu2 is coordinated by the second pyridyl-N, hydrazine-N and the second hydrazine-N of the first hydrazine group (Fig. 1 ).The coordination environments of both Cu^II^ ions are completed by one chloride ion and one methaol molecule. Both of the copper atoms adopt similar distorted 4 + 1 coordinated square-pyramid geometries with a distortion parameter 0.197 for Cu1 and 0.101 for Cu2 (Addison *et al.*, 1984). The crystal packing is stabilised by O-H···O(perchlorate), O-H···Cl and N-H···Cl hydrogen bonding (Fig. 2) The dihedral angle between the pyridyl groups is 27.46 (10)°.

### Experimental

A methanolic solution (15 ml) containing the ligand (0.1 mmol, 0.03 g) was added dropwise to a methanolic solution (10 ml) containing CuCl_2_.2H_2_O (0.1 mmol, 0.034 g). After stirring for 2 h, the solution was filtered. Dark-green block-shaped crystals suitable for single-crystal X-ray diffraction were obtained by evaporating the resulting filtration in air for several days (yield: 65.6% based on the ligand). Anal calc (%). for C_17_H_23_Cl_3_Cu_2_N_6_O_7_: H 3.53 C 31.09 N 12.79 Found: H 3.42, C 31.21, N 12.86.

### Refinement

C-bound H atoms were placed geometrically and allowed to ride during refinement with C—H = 0.93–0.96 Å with *U*_iso_(H) = 1.2 U_eq_(C). O-bound H atoms were located in a difference Fourier map and refined as riding with the parent atom with an isotropic thermal parameter 1.5 times that of the parent atom.

### Crystal data

**Table:**

[Cu_2_(C_15_H_15_N_6_O)Cl_2_(CH_4_O)_2_]ClO_4_	*F*(000) = 2656
*M**_r_* = 656.84	*D*_x_ = 1.744 Mg m^−^^3^
Orthorhombic, *P**b**c**a*	Mo *K*α radiation, λ = 0.71073 Å
Hall symbol: -P 2ac 2ab	Cell parameters from 15443 reflections
*a* = 8.0319 (3) Å	θ = 2.3–27.0°
*b* = 16.6784 (6) Å	µ = 2.07 mm^−^^1^
*c* = 37.3492 (13) Å	*T* = 298 K
*V* = 5003.3 (3) Å^3^	Block, dark-green
*Z* = 8	0.18 × 0.16 × 0.12 mm

### Data collection

**Table:**

Bruker SMART APEXII CCD area-detector diffractometer	5692 independent reflections
Radiation source: fine-focus sealed tube	4016 reflections with *I* > 2σ(*I*)
graphite	*R*_int_ = 0.064
φ and ω scans	θ_max_ = 27.5°, θ_min_ = 2.2°
Absorption correction: multi-scan (*SHELXTL*; Sheldrick, 2008)	*h* = −10→10
*T*_min_ = 0.675, *T*_max_ = 0.783	*k* = −21→21
30236 measured reflections	*l* = −48→39

### Refinement

**Table:**

Refinement on *F*^2^	Primary atom site location: structure-invariant direct methods
Least-squares matrix: full	Secondary atom site location: difference Fourier map
*R*[*F*^2^ > 2σ(*F*^2^)] = 0.048	Hydrogen site location: inferred from neighbouring sites
*wR*(*F*^2^) = 0.142	H-atom parameters constrained
*S* = 1.04	*w* = 1/[σ^2^(*F*_o_^2^) + (0.0713*P*)^2^ + 3.3159*P*] where *P* = (*F*_o_^2^ + 2*F*_c_^2^)/3
5692 reflections	(Δ/σ)_max_ = 0.001
318 parameters	Δρ_max_ = 0.90 e Å^−^^3^
0 restraints	Δρ_min_ = −0.67 e Å^−^^3^

### Special details

**Table:**

Geometry. All esds (except the esd in the dihedral angle between two l.s. planes) are estimated using the full covariance matrix. The cell esds are taken into account individually in the estimation of esds in distances, angles and torsion angles; correlations between esds in cell parameters are only used when they are defined by crystal symmetry. An approximate (isotropic) treatment of cell esds is used for estimating esds involving l.s. planes.
Refinement. Refinement of *F*^2^ against ALL reflections. The weighted *R*-factor *wR* and goodness of fit *S* are based on *F*^2^, conventional *R*-factors *R* are based on *F*, with *F* set to zero for negative *F*^2^. The threshold expression of *F*^2^ > σ(*F*^2^) is used only for calculating *R*-factors(gt) *etc*. and is not relevant to the choice of reflections for refinement. *R*-factors based on *F*^2^ are statistically about twice as large as those based on *F*, and *R*- factors based on ALL data will be even larger.

### Fractional atomic coordinates and isotropic or equivalent isotropic displacement parameters (Å^2^)

**Table:**

	*x*	*y*	*z*	*U*_iso_*/*U*_eq_	
Cu1	0.62011 (6)	0.28060 (3)	0.033586 (13)	0.03826 (15)	
Cu2	0.81104 (6)	0.39647 (3)	0.143708 (12)	0.03630 (15)	
C1	0.8701 (6)	0.4718 (3)	0.21601 (12)	0.0538 (11)	
H1A	0.8464	0.5205	0.2050	0.065*	
C1W	0.3072 (9)	0.2671 (5)	0.08697 (17)	0.112 (3)	
H1WA	0.1939	0.2822	0.0916	0.167*	
H1WB	0.3761	0.2840	0.1066	0.167*	
H1WC	0.3143	0.2100	0.0844	0.167*	
C2	0.9166 (7)	0.4715 (3)	0.25190 (13)	0.0610 (13)	
H2A	0.9248	0.5191	0.2647	0.073*	
C2W	1.2080 (7)	0.4315 (5)	0.1542 (2)	0.098 (2)	
H2WA	1.3027	0.4607	0.1455	0.147*	
H2WB	1.2250	0.3752	0.1504	0.147*	
H2WC	1.1941	0.4417	0.1794	0.147*	
C3	0.9502 (6)	0.3989 (3)	0.26802 (12)	0.0569 (12)	
H3A	0.9802	0.3970	0.2921	0.068*	
C4	0.9391 (5)	0.3293 (3)	0.24833 (11)	0.0486 (10)	
H4A	0.9623	0.2799	0.2588	0.058*	
C5	0.8929 (5)	0.3338 (3)	0.21264 (11)	0.0411 (9)	
C6	0.8894 (7)	0.1789 (3)	0.20249 (13)	0.0591 (12)	
H6A	0.8749	0.1424	0.1829	0.089*	
H6B	0.8048	0.1694	0.2201	0.089*	
H6C	0.9971	0.1708	0.2130	0.089*	
C7	0.8762 (5)	0.2626 (3)	0.18923 (11)	0.0398 (9)	
C8	0.7643 (5)	0.2622 (2)	0.09818 (10)	0.0368 (8)	
C9	0.8716 (5)	0.4960 (3)	0.06034 (12)	0.0481 (10)	
H9A	0.9033	0.4835	0.0845	0.072*	
H9B	0.8118	0.5459	0.0600	0.072*	
H9C	0.9695	0.5006	0.0457	0.072*	
C10	0.7642 (4)	0.4315 (2)	0.04617 (10)	0.0346 (8)	
C11	0.6971 (5)	0.4355 (2)	0.00899 (10)	0.0364 (8)	
C12	0.7161 (5)	0.5001 (3)	−0.01319 (11)	0.0447 (10)	
H12A	0.7704	0.5459	−0.0051	0.054*	
C13	0.6538 (6)	0.4967 (3)	−0.04776 (13)	0.0543 (12)	
H13A	0.6684	0.5398	−0.0633	0.065*	
C14	0.5709 (6)	0.4299 (3)	−0.05879 (12)	0.0579 (12)	
H14A	0.5266	0.4270	−0.0818	0.069*	
C15	0.5539 (6)	0.3668 (3)	−0.03538 (11)	0.0491 (10)	
H15A	0.4969	0.3213	−0.0429	0.059*	
N1	0.8586 (4)	0.4049 (2)	0.19707 (9)	0.0411 (8)	
N2	0.8459 (4)	0.28245 (19)	0.15635 (9)	0.0387 (7)	
N3	0.8162 (5)	0.2289 (2)	0.12969 (9)	0.0424 (8)	
H3B	0.8291	0.1781	0.1324	0.051*	
N4	0.7825 (4)	0.34270 (19)	0.09563 (8)	0.0359 (7)	
N5	0.7277 (4)	0.36484 (18)	0.06188 (8)	0.0340 (7)	
N6	0.6157 (4)	0.3682 (2)	−0.00234 (9)	0.0392 (7)	
O1	0.7099 (4)	0.21631 (15)	0.07431 (7)	0.0447 (7)	
O1W	0.3611 (4)	0.3035 (2)	0.05585 (9)	0.0620 (9)	
O2W	1.0641 (4)	0.45651 (19)	0.13568 (8)	0.0540 (8)	
O4	0.2605 (8)	0.6862 (8)	0.1635 (3)	0.276 (6)	
O5	0.1217 (13)	0.6334 (4)	0.20860 (15)	0.189 (4)	
O6	0.0314 (10)	0.7448 (4)	0.17370 (16)	0.159 (3)	
O7	0.0200 (11)	0.6230 (3)	0.15081 (16)	0.167 (3)	
Cl1	0.54972 (14)	0.18014 (6)	−0.00266 (3)	0.0493 (3)	
Cl2	0.64899 (14)	0.50485 (7)	0.13951 (3)	0.0529 (3)	
Cl3	0.1126 (2)	0.66852 (9)	0.17526 (4)	0.0740 (4)	
H1W	0.2879	0.2904	0.0347	0.089*	
H2W	1.0197	0.5118	0.1398	0.089*	

### Atomic displacement parameters (Å^2^)

**Table:**

	*U*^11^	*U*^22^	*U*^33^	*U*^12^	*U*^13^	*U*^23^
Cu1	0.0495 (3)	0.0282 (3)	0.0371 (3)	−0.0014 (2)	−0.0063 (2)	−0.00165 (19)
Cu2	0.0432 (3)	0.0324 (3)	0.0332 (3)	0.0015 (2)	−0.00304 (19)	−0.00154 (19)
C1	0.063 (3)	0.047 (3)	0.051 (3)	−0.004 (2)	−0.004 (2)	−0.006 (2)
C1W	0.101 (5)	0.180 (8)	0.055 (4)	−0.031 (5)	0.007 (4)	0.014 (4)
C2	0.075 (3)	0.060 (3)	0.048 (3)	−0.006 (3)	−0.007 (2)	−0.013 (2)
C2W	0.051 (3)	0.128 (6)	0.115 (5)	0.007 (4)	−0.011 (3)	−0.010 (5)
C3	0.062 (3)	0.072 (4)	0.037 (2)	−0.005 (3)	−0.005 (2)	−0.004 (2)
C4	0.054 (2)	0.057 (3)	0.034 (2)	−0.004 (2)	−0.0024 (19)	0.0016 (19)
C5	0.038 (2)	0.048 (3)	0.037 (2)	−0.0022 (17)	0.0008 (16)	0.0019 (19)
C6	0.082 (3)	0.045 (3)	0.050 (3)	0.006 (2)	−0.002 (2)	0.007 (2)
C7	0.042 (2)	0.040 (2)	0.038 (2)	0.0016 (17)	0.0020 (17)	0.0037 (17)
C8	0.047 (2)	0.032 (2)	0.0311 (19)	−0.0011 (16)	0.0009 (16)	−0.0001 (16)
C9	0.055 (2)	0.040 (2)	0.050 (3)	−0.0123 (19)	−0.0035 (19)	0.0011 (19)
C10	0.0360 (19)	0.030 (2)	0.038 (2)	0.0001 (15)	0.0023 (16)	−0.0010 (16)
C11	0.0362 (19)	0.033 (2)	0.040 (2)	0.0052 (16)	0.0035 (16)	−0.0015 (16)
C12	0.051 (2)	0.037 (2)	0.046 (2)	0.0006 (18)	0.0006 (19)	0.0029 (18)
C13	0.067 (3)	0.049 (3)	0.047 (3)	0.013 (2)	0.002 (2)	0.015 (2)
C14	0.076 (3)	0.057 (3)	0.041 (2)	0.011 (3)	−0.013 (2)	0.002 (2)
C15	0.060 (3)	0.042 (2)	0.045 (2)	0.003 (2)	−0.015 (2)	0.0004 (19)
N1	0.0473 (19)	0.037 (2)	0.0389 (18)	−0.0001 (14)	−0.0046 (15)	−0.0018 (15)
N2	0.0471 (19)	0.0336 (18)	0.0355 (17)	−0.0011 (14)	−0.0040 (14)	−0.0013 (14)
N3	0.065 (2)	0.0291 (18)	0.0333 (17)	0.0030 (15)	−0.0065 (15)	0.0006 (14)
N4	0.0467 (18)	0.0319 (18)	0.0289 (16)	−0.0031 (14)	−0.0004 (13)	0.0008 (13)
N5	0.0393 (17)	0.0304 (17)	0.0322 (16)	0.0006 (13)	−0.0007 (13)	−0.0013 (13)
N6	0.0456 (18)	0.0320 (18)	0.0399 (18)	0.0011 (14)	−0.0058 (14)	−0.0006 (14)
O1	0.0696 (19)	0.0259 (14)	0.0386 (15)	−0.0030 (13)	−0.0103 (13)	−0.0012 (12)
O1W	0.060 (2)	0.069 (2)	0.057 (2)	0.0002 (16)	0.0050 (16)	0.0026 (17)
O2W	0.0486 (17)	0.055 (2)	0.0578 (19)	−0.0072 (15)	−0.0076 (14)	−0.0021 (15)
O4	0.077 (4)	0.488 (17)	0.263 (9)	−0.028 (7)	−0.029 (5)	0.232 (11)
O5	0.361 (11)	0.138 (5)	0.068 (3)	−0.100 (6)	−0.028 (5)	0.020 (3)
O6	0.242 (8)	0.129 (5)	0.108 (4)	0.047 (6)	−0.015 (5)	−0.033 (4)
O7	0.295 (9)	0.073 (3)	0.131 (5)	−0.026 (4)	−0.096 (5)	−0.015 (3)
Cl1	0.0617 (7)	0.0353 (6)	0.0509 (6)	−0.0058 (5)	−0.0110 (5)	−0.0075 (4)
Cl2	0.0561 (6)	0.0500 (7)	0.0526 (6)	0.0180 (5)	−0.0067 (5)	−0.0051 (5)
Cl3	0.0885 (10)	0.0661 (9)	0.0673 (9)	−0.0155 (7)	0.0015 (7)	−0.0110 (7)

### Geometric parameters (Å, °)

**Table:**

Cu1—N5	1.959 (3)	C6—H6B	0.9600
Cu1—N6	1.985 (3)	C6—H6C	0.9600
Cu1—O1	1.996 (3)	C7—N2	1.294 (5)
Cu1—Cl1	2.2270 (11)	C8—O1	1.253 (4)
Cu1—O1W	2.273 (3)	C8—N4	1.354 (5)
Cu2—N2	1.979 (3)	C8—N3	1.367 (5)
Cu2—N4	2.020 (3)	C9—C10	1.477 (5)
Cu2—N1	2.034 (3)	C9—H9A	0.9600
Cu2—Cl2	2.2330 (11)	C9—H9B	0.9600
Cu2—O2W	2.285 (3)	C9—H9C	0.9600
C1—N1	1.324 (6)	C10—N5	1.292 (5)
C1—C2	1.392 (6)	C10—C11	1.491 (5)
C1—H1A	0.9300	C11—N6	1.366 (5)
C1W—O1W	1.380 (7)	C11—C12	1.367 (6)
C1W—H1WA	0.9600	C12—C13	1.386 (6)
C1W—H1WB	0.9600	C12—H12A	0.9300
C1W—H1WC	0.9600	C13—C14	1.362 (7)
C2—C3	1.378 (7)	C13—H13A	0.9300
C2—H2A	0.9300	C14—C15	1.375 (6)
C2W—O2W	1.411 (6)	C14—H14A	0.9300
C2W—H2WA	0.9600	C15—N6	1.330 (5)
C2W—H2WB	0.9600	C15—H15A	0.9300
C2W—H2WC	0.9600	N2—N3	1.358 (5)
C3—C4	1.378 (6)	N3—H3B	0.8600
C3—H3A	0.9300	N4—N5	1.386 (4)
C4—C5	1.386 (6)	O1W—H1W	1.0086
C4—H4A	0.9300	O2W—H2W	1.0003
C5—N1	1.349 (5)	O4—Cl3	1.301 (7)
C5—C7	1.481 (6)	O5—Cl3	1.378 (6)
C6—C7	1.486 (6)	O6—Cl3	1.430 (7)
C6—H6A	0.9600	O7—Cl3	1.401 (5)
			
N5—Cu1—N6	81.04 (13)	O1—C8—N3	118.1 (4)
N5—Cu1—O1	79.33 (12)	N4—C8—N3	115.5 (3)
N6—Cu1—O1	156.56 (13)	C10—C9—H9A	109.5
N5—Cu1—Cl1	168.39 (10)	C10—C9—H9B	109.5
N6—Cu1—Cl1	97.97 (10)	H9A—C9—H9B	109.5
O1—Cu1—Cl1	98.68 (8)	C10—C9—H9C	109.5
N5—Cu1—O1W	94.93 (13)	H9A—C9—H9C	109.5
N6—Cu1—O1W	96.16 (13)	H9B—C9—H9C	109.5
O1—Cu1—O1W	98.17 (13)	N5—C10—C9	126.7 (4)
Cl1—Cu1—O1W	96.68 (10)	N5—C10—C11	112.3 (3)
N2—Cu2—N4	78.56 (13)	C9—C10—C11	120.8 (3)
N2—Cu2—N1	78.81 (14)	N6—C11—C12	120.8 (4)
N4—Cu2—N1	157.34 (13)	N6—C11—C10	115.2 (3)
N2—Cu2—Cl2	151.28 (11)	C12—C11—C10	124.0 (4)
N4—Cu2—Cl2	103.35 (10)	C11—C12—C13	119.5 (4)
N1—Cu2—Cl2	97.03 (10)	C11—C12—H12A	120.2
N2—Cu2—O2W	109.06 (13)	C13—C12—H12A	120.2
N4—Cu2—O2W	100.30 (12)	C14—C13—C12	119.4 (4)
N1—Cu2—O2W	86.07 (13)	C14—C13—H13A	120.3
Cl2—Cu2—O2W	98.88 (9)	C12—C13—H13A	120.3
N1—C1—C2	122.0 (5)	C13—C14—C15	118.9 (4)
N1—C1—H1A	119.0	C13—C14—H14A	120.6
C2—C1—H1A	119.0	C15—C14—H14A	120.6
O1W—C1W—H1WA	109.5	N6—C15—C14	122.6 (4)
O1W—C1W—H1WB	109.5	N6—C15—H15A	118.7
H1WA—C1W—H1WB	109.5	C14—C15—H15A	118.7
O1W—C1W—H1WC	109.5	C1—N1—C5	119.7 (4)
H1WA—C1W—H1WC	109.5	C1—N1—Cu2	126.5 (3)
H1WB—C1W—H1WC	109.5	C5—N1—Cu2	113.6 (3)
C3—C2—C1	118.5 (5)	C7—N2—N3	124.1 (3)
C3—C2—H2A	120.8	C7—N2—Cu2	119.9 (3)
C1—C2—H2A	120.8	N3—N2—Cu2	115.6 (2)
O2W—C2W—H2WA	109.5	N2—N3—C8	114.7 (3)
O2W—C2W—H2WB	109.5	N2—N3—H3B	122.6
H2WA—C2W—H2WB	109.5	C8—N3—H3B	122.6
O2W—C2W—H2WC	109.5	C8—N4—N5	107.1 (3)
H2WA—C2W—H2WC	109.5	C8—N4—Cu2	113.0 (2)
H2WB—C2W—H2WC	109.5	N5—N4—Cu2	136.6 (2)
C4—C3—C2	119.6 (4)	C10—N5—N4	124.8 (3)
C4—C3—H3A	120.2	C10—N5—Cu1	118.2 (3)
C2—C3—H3A	120.2	N4—N5—Cu1	116.1 (2)
C3—C4—C5	119.0 (4)	C15—N6—C11	118.7 (4)
C3—C4—H4A	120.5	C15—N6—Cu1	128.4 (3)
C5—C4—H4A	120.5	C11—N6—Cu1	112.8 (2)
N1—C5—C4	121.2 (4)	C8—O1—Cu1	109.9 (2)
N1—C5—C7	115.5 (3)	C1W—O1W—Cu1	121.4 (4)
C4—C5—C7	123.3 (4)	C1W—O1W—H1W	112.5
C7—C6—H6A	109.5	Cu1—O1W—H1W	102.2
C7—C6—H6B	109.5	C2W—O2W—Cu2	122.3 (4)
H6A—C6—H6B	109.5	C2W—O2W—H2W	119.3
C7—C6—H6C	109.5	Cu2—O2W—H2W	93.8
H6A—C6—H6C	109.5	O4—Cl3—O5	110.7 (5)
H6B—C6—H6C	109.5	O4—Cl3—O7	112.7 (7)
N2—C7—C5	111.9 (4)	O5—Cl3—O7	112.7 (4)
N2—C7—C6	124.7 (4)	O4—Cl3—O6	101.6 (6)
C5—C7—C6	123.4 (4)	O5—Cl3—O6	116.0 (5)
O1—C8—N4	126.4 (3)	O7—Cl3—O6	102.3 (4)

### Hydrogen-bond geometry (Å, °)

**Table:**

*D*—H···*A*	*D*—H	H···*A*	*D*···*A*	*D*—H···*A*
N3—H3B···Cl2^i^	0.86	2.91	3.766 (4)	176
O1W—H1W···Cl1^ii^	1.01	2.31	3.205 (4)	147
O2W—H2W···O7^iii^	1.00	1.90	2.855 (6)	159

Symmetry codes: (i) −*x*+3/2, *y*−1/2, *z*; (ii) *x*−1/2, −*y*+1/2, −*z*; (iii) *x*+1, *y*, *z*.
